# Evolutionary conservation and functional roles of ncRNA

**DOI:** 10.3389/fgene.2012.00205

**Published:** 2012-10-09

**Authors:** Zhipeng Qu, David L. Adelson

**Affiliations:** School of Molecular and Biomedical Science, The University of AdelaideAdelaide, SA, Australia

**Keywords:** ncRNA, transcription, genetic, long ncRNA, evolution, molecular, gene regulation

## Abstract

Non-coding RNAs (ncRNAs) are a class of transcribed RNA molecules without protein-coding potential. They were regarded as transcriptional noise, or the byproduct of genetic information flow from DNA to protein for a long time. However, in recent years, a number of studies have shown that ncRNAs are pervasively transcribed, and most of them show evidence of evolutionary conservation, although less conserved than protein-coding genes. More importantly, many ncRNAs have been confirmed as playing crucial regulatory roles in diverse biological processes and tumorigenesis. Here we summarize the functional significance of this class of “dark matter” in terms its genomic organization, evolutionary conservation, and broad functional classes.

## INTRODUCTION

As the basis of genetics, the “central dogma” describes the genetic information flow of life (Crick, 1970). The functional roles of DNA as the repository genetic information, and protein as the functional incarnation of that information, have been viewed as the dominant molecular roles in the cell for nearly four decades, while RNA was subordinated as a temporary intermediate of this information flow. However, the hypothesis of an “RNA world” proposed by Gilbert (1986) challenged the “central dogma” view of the biological role of RNA. The RNA world theory proposed that the origin of life is based on RNA, which could both store genetic data and carry out functions such as catalysis. Although the RNA world hypothesis is debated, a hidden “RNA regulatory world” has been proposed in recent studies describing non-coding RNAs (ncRNAs). Thousands of pervasively transcribed ncRNAs have been identified in human, mouse, and other species. Furthermore, these ncRNAs also show clear evolutionary conservation. Many ncRNAs, especially recently identified long ncRNAs, have been shown to play key regulatory roles in diverse biological processes, including pathological processes such as tumorigenesis.

## DEFINITION AND CLASSIFICATION OF ncRNAs

Previously ncRNA has been referred to by various names: non-protein-coding RNAs (npcRNAs; Mattick, 2003), intronic and intergenic ncRNAs (Louro et al., 2009), and mRNAs-like ncRNAs (Inagaki et al., 2005; Rymarquis et al., 2008). At present, ncRNAs are classified on the basis of their main functions: structural ncRNAs and regulatory ncRNAs (Mattick and Makunin, 2006). Structural ncRNAs include transfer RNAs (tRNAs), ribosomal RNAs (rRNAs), spliceosomal uRNAs (snRNAs), and snoRNAs. Most of these ncRNAs have well-established structural functions. ncRNAs with regulatory roles in gene expression are classified as regulatory ncRNAs, including small interfering RNA (siRNA), micro-RNAs (miRNAs), piwi-RNAs (piRNAs), long ncRNAs, and long intergenic ncRNAs.

Numerous studies in the past decade have focused on small ncRNAs. As a result of these studies it is now clear that this class of ncRNAs regulates almost every aspect of gene expression (Goodrich and Kugel, 2006).

In addition to small ncRNAs, large numbers of long ncRNAs have recently been revealed by large-scale transcriptome analyses. Although only a limited number of long ncRNAs have well-characterized structures and functions, many studies suggest that this class of ncRNA accounts for a large fraction of the transcriptome, and they are believed to play important roles in many key molecular regulatory processes (Yazgan and Krebs, 2007; Umlauf et al., 2008; Mercer et al., 2009). We will recapitulate the pervasive transcription and genome/transcriptome complexity of these regulatory ncRNAs, particularly with respect to long ncRNAs, and review their primary proposed functional models.

## PERVASIVE TRANSCRIPTION OF ncRNAs

Rapid development in analytical technologies, such as whole genome tilling arrays, capped analysis of gene expression (CAGE), Chip-chip, Chip-seq, and RNA deep sequencing have revised people’s views of eukaryotic genome/transcriptome complexity (Carninci, 2006; Gustincich et al., 2006). In the past decade, large-scale transcriptome analyses of several organisms indicate that genomes are pervasively transcribed and ncRNAs account for a large proportion of the whole transcriptome (Bertone et al., 2004; Birney et al., 2007).

### THE HUMAN TRANSCRIPTOME IS MORE COMPLICATED THAN EXPECTED

It has been more than decade since the human genome was sequenced, yet the decoding of this information is far from complete. According to the statistics of the version 34b of the Ensembl Human Genome, there are about 20–25,000 protein-coding genes, with a total coding length of ~34 Mb, which only occupies ~1.2% of the whole genome. On the other hand, about 1,679 Mb non-coding sequences, accounting for more than half (~57%) of the whole human genome, are believed to be transcribed. These non-coding sequences include introns, untranslated regions (UTRs), and other intronic and exonic sequences covered by spliced cDNAs/ESTs that are not annotated as protein coding. The 47:1 ratio of transcribed non-coding regions to coding regions indicates that ncRNAs represent a large share of the human transcriptome (Frith et al., 2005). Tiling array and other several large-scale analyses of the human genome have also provided strong support to this hypothesis. The large-scale transcriptional analysis of human chromosome 21 and 22 using oligonucleotide arrays showed that only 2.6% (26,516 of 1,011,768) probe pairs that interrogate approximately 35 Mb non-repetitive regions of these two chromosomes are detected inside the annotated exons of well-characterized genes. Ninety-four percent of the probes are expressed and located outside annotated exons in 1 of 11 detected cell lines, and the percentage is 88 for 5 of 11 cell lines. This indicates that some of non-coding transcripts are cell type-specific expressed (Kapranov et al., 2002). Further in-depth transcriptome analysis of human chromosome 21 and 22 provided similar results: nearly half of the studied transcripts originated outside of well-annotated exons, and these novel transcripts seem to have less variation and be cell type-specific in expression compared to well-characterized genes (Kampa et al., 2004). These results are reinforced by subsequent high-density genome tiling array studies of 10 human chromosomes (Cheng et al., 2005) and massively parallel signature sequencing (MPSS) analysis (Jongeneel et al., 2005). All of these results clearly demonstrate that the human genome is highly transcribed and the landscape of human ncRNAs is extremely complex.

### ncRNAs ARE A MAJOR COMPONENT OF THE MOUSE TRANSCRIPTOME

Large-scale transcription analyses of the mouse genome have also revealed that ncRNAs are commonly transcribed. Early in 2002, a dataset of ncRNAs from the mouse transcriptome was proposed based on functional annotation of full-length cDNAs (also called FANTOM2). Over one-third (34.9%) of 33,409 “transcriptional units,” clustered from 60,770 full-length cDNAs, were predicted as novel non-coding transcripts (Okazaki et al., 2002). According to the analysis of FANTOM3 in 2006, the number of predicted distinct non-coding transcripts had increased to 34,030, over threefold compared to FANTOM2 (Maeda et al., 2006). Further analysis of FANTOM3 by the FANTOM Consortium revealed that many putative ncRNAs were singletons in the full-length cDNA set but that 3,652 cDNAs, which were supported by overlapping with both the initiation and termination sites of ESTs, CAGE tags, or other cDNA clones, were identified as ncRNAs. In addition, 3,012 cDNAs that were previously regarded as truncated CDS were identified as genuine transcripts and were believed to be the ncRNA variants of protein-coding cDNAs (Carninci et al., 2005). Transcriptome sequencing of mouse embryonic stem cells also revealed 1,022 non-coding expressed transcripts, and some of them were shown to have expression levels correlated with differentiation state (Araki et al., 2006). The existence of large numbers of ncRNAs transcribed from the mouse genome was subsequently validated by RT-PCR, microarray, and northern blot analyses (Ravasi et al., 2006).

### OTHER SPECIES ALSO EXPRESS LARGE NUMBERS OF ncRNAs

Although there have been fewer large-scale transcriptome studies of species other than human and mouse, they have confirmed the existence of ncRNAs. Seventeen distinct non-protein-coding polyadenylated transcripts were identified from the intergenic regions of the fly genome (Tupy et al., 2005). Moreover, 136 strong candidates for mRNA-like ncRNAs were screened from 11,691 fly full-length cDNAs, and 35 of them were expressed during embryogenesis. Of these 35 mRNA-like ncRNAs, 27 were detected only in specific tissues (Inagaki et al., 2005). These results indicate that many mRNA-like ncRNAs are expected to play important roles in the fly. In 2005, approximately 1,300 genes that produce functional ncRNAs were demonstrated in the worm *C. elegans* (Stricklin et al., 2005). However, the worm transcriptome is much more complicated than expected. The worm non-coding transcriptome mapped by whole-genome tiling array showed that at least 70% of the total worm genome was transcribed, and 44% of the total observed transcriptional output on the array was predicted to consist of non-polyadenylated transcripts without protein-coding potential. Seventy percent of these non-polyadenylated transcripts were shown to overlap with the coordinates of coding loci in complicated fashions (He et al., 2007). The prevalence of ncRNAs extends even further, as studies of *Saccharomyces cerevisiae* have also revealed large numbers of ncRNAs (Havilio et al., 2005; Miura et al., 2006).

### EVIDENCE FROM WELL-CHARACTERIZED LONG ncRNA DATASETS

In past several years, our knowledge of long ncRNAs has been expanding thanks to the identification and annotation of diverse classes of long ncRNAs from human, mouse, and other species (Table 1). About 1,600 large intervening/intergenic ncRNAs (lincRNAs) were identified based on the chromatin-state maps from four mouse cell types (Guttman et al., 2009). Based on the same method, ~3,300 lincRNAs were characterized according to the chromatin-state maps of various human cell types (Khalil et al., 2009). Moreover, a class of ~3,200 enhancer-like long ncRNAs were discovered as a result of the ENCODE project (Orom et al., 2010). The rapid drop in price of next generation sequencing drove the generation of large amounts of RNA-seq data from a number of species. More than a thousand multi-exonic lincRNAs were revealed by reconstruction of transcriptomes from three mouse cell types (Guttman et al., 2010). Human transcriptome data from more sources (24 tissues and cell types), allowed the reconstruction of more than 8,000 human lincRNAs (Cabili et al., 2011). Large numbers of long ncRNAs were also found in zebrafish, fly, and worm transcriptomes based on RNA-seq data. A stringent set of 1,133 non-coding multi-exonic transcripts, including lincRNAs, intronic overlapping long ncRNAs, exonic antisense overlapping long ncRNAs, and precursors for small RNAs (sRNAs), were identified from transcriptome data of eight early zebrafish development stages (Pauli et al., 2011). Recently, 1,199 putative lincRNAs and more than 800 lincRNAs were annotated from fly and worm transcriptomes based on RNA-seq data (Nam and Bartel, 2012; Young et al., 2012).

**Table 1:** Recently well-characterized long ncRNA datasets.

Dataset	Number of Source ncRNAs	Method	Reference
Chromatin-state-based lincRNAs (human)	4,860*	10 cell types	Chromatin signature identification (K4–K36 domain)	Khalil et al. (2009)
Enhancer-like long ncRNAs (human)	3,011	Multiple	Screening from GENCODE annotation	Orom et al. (2010)
RNA-seq-based lincRNAs (human)	8,195	24 tissues and cell types	Screening from assembled RNA-seq data	Cabili et al. (2011)
Chromatin-state-based lincRNAs (mouse)	2,127*	Four cell types	Chromatin signature identification (K4–K36 domain)	Guttman et al. (2009)
RNA-seq-based lincRNAs (mouse)	1,140	Three cell types	Screening from assembled RNA-seq data	Guttman et al. (2010)
RNA-seq-based long ncRNAs (zebrafish)	1,133	Eight embryonic stages	Screening from assembled RNA-seq data	Pauli et al. (2011)
RNA-seq-based lincRNAs (fruit fly)	1,119	30 developmental time points	Screening from assembled RNA-seq data	Young et al. (2012)
RNA-seq-based lincRNAs (*C. elegans*)	882	Multiple	Screening from assembled RNA-seq data	Nam and Bartel (2012)

* These are the exons identified by microarray from non-coding k4–k36 domains.

## EVOLUTIONARY CONSERVATION OF LONG ncRNAs

In contrast to well-conserved small ncRNAs, like miRNAs, the evolutionary sequence conservation of long ncRNAs is less pronounced. Most studies have shown that long ncRNAs are poorly conserved compared to protein-coding genes (Louro et al., 2009; Mercer et al., 2009). In a comparison between human and mouse long ncRNAs, Pang et al. (2006) found that the sequence homology of long ncRNAs was similar to that of introns (<70% between mice and humans) and a little less conserved than 5′ or 3′ UTRs. Thus the evolutionary constraints acting on long ncRNAs may differ from the constraints affecting small ncRNAs, allowing long ncRNAs to evolve faster than small RNAs. However, conservation analysis of long ncRNAs based on 50-nt window size revealed that many long ncRNAs may retain patches of higher conservation within their overall sequences, possibly representing interaction sites with RNA-binding proteins (Pang et al., 2006).

Recently, novel long ncRNA datasets identified from diverse species have confirmed that most long ncRNAs are less conserved than protein-coding genes while still showing clear conservation. Over 95% of the 1,600 mouse lincRNAs identified by chromatin-state maps showed clear evolutionary conservation (Guttman et al., 2009). Subsequent analysis of 3,300 human chromatin-state based lincRNAs also indicated that these lincRNAs were more conserved than intronic regions (Khalil et al., 2009). Analysis of human enhancer-like long ncRNAs also showed that the global conservation levels of these long ncRNAs were less than protein-coding genes, but higher than ancestral repeats (Orom et al., 2010). Long ncRNAs reconstructed from mouse RNA-seq data showed similar conservation levels compared to chromatin-state based lincRNAs (Guttman et al., 2010). In human, RNA-seq based long ncRNAs showed moderate conservation across different species (Cabili et al., 2011). The conservation of zebrafish RNA-seq derived long ncRNAs assessed by CBL score was substantially lower than protein-coding genes and comparable to intronic sequences (Pauli et al., 2011). Analysis from the fly RNA-seq based lincRNAs also showed that most of these ncRNAs, even for those expressed at low levels, have significantly lower nucleotide substitution rates compared with either untranscribed intergenic sequence or neutrally evolving short introns (Young et al., 2012). RNA-seq based lincRNAs identified from another invertebrate organism *C. elegans* were differentiated into two subclasses according to their conservation, non-conserved and moderately conserved. Similar to vertebrates, some of these *C. elegans* lincRNAs also tend to have short regions of conservation (Nam and Bartel, 2012).

Overall, while long ncRNAs identified from different species and based on different methods showed slightly different levels of conservation, it is clear that long ncRNAs are less conserved than protein-coding genes but still exhibit clear conservation compared to non-functional genomic elements. One widely accepted interpretation of poor sequence conservation for long ncRNAs is that long ncRNAs may function at the secondary structure level instead of the primary sequence level. This is in contrast to protein-coding, which genes require conserved nucleotide sequence to encode higher levels of structure with similar biological functions. Differently, the small conserved patches observed in some long ncRNAs might be sufficient to support the functions of these long ncRNAs, by binding with proteins, interacting with DNA promoters or with UTRs of mRNAs. Finally, the long ncRNA datasets described above were identified using different methods, possibly fostering bias for some classes of long ncRNAs, which might be subject to different selective pressure.

## GENOMIC ORGANIZATION OF ncRNAs

**FIGURE 1:**
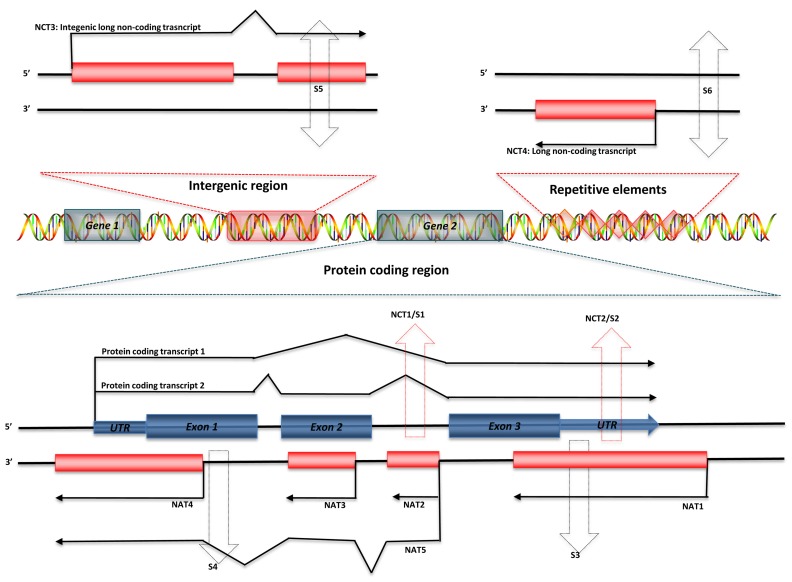
**Genomic organization of regulatory ncRNAs in mammalian genome.** Green symbols represent protein-coding transcripts; red symbols represent non-coding transcripts; black dashed arrows represent small ncRNAs; and red dashed arrows represent shared regions transcribed as long ncRNAs or small ncRNAs. Long ncRNAs can be transcribed from: (1) Non-coding regions of protein-coding transcript: intron – NCT1; UTRs – NCT2. (2) Antisense of protein-coding transcripts: convergent (tail–tail) antisense transcript – NAT1; intronic antisense transcript – NAT2; contained antisense transcript – NAT3; divergent (head–head) antisense transcript – NAT4; mixed-model antisense transcript – NAT5. (3) Intergenic region: NCT3. (4) Repetitive elements: NCT4. Small ncRNAs can be transcribed from introns (S1) or UTRs (S2) of protein-coding genes, antisense region of UTRs (S3) or exons (S4), both strands of intergenic regions (S5), and both strands of repetitive elements (S6).

Regulatory ncRNAs originate from different genomic regions (**Figure 1**). UTRs account for many of the regions encoding ncRNAs. Statistics from the UCSC human genome (NCBI build 35) show that total UTR sequences account for ~1.1% of the whole human genome, nearly equivalent in length to protein-coding regions (32–34 Mb; Frith et al., 2005). This suggests that there may be unknown regulatory elements in these regions. Studies using CAGE, serial analysis of gene expression (SAGE), cDNA libraries, and microarray expression profiles have shown that there are independent transcripts expressed from 3′ UTRs. This class of independent transcripts has been termed “uaRNAs” (UTR-associated RNAs), some of which have been validated as being expressed in cell- and subcellular-specific fashion (Mercer et al., 2010). In addition to UTRs, other non-coding regions of genome, such as intronic sequences are also a potential source of functional ncRNAs. Over 30% of the human genome is made up of intronic sequences (Mattick and Gagen, 2001), and many highly conserved sequences have been identified in introns (Taft et al., 2007). Recent research has indicated that there are a large number of long intronic ncRNAs in both human and mouse (Nakaya et al., 2007; Louro et al., 2008,^2009^). Long ncRNAs can also be derived from both the sense and antisense strands of various genomic regions, some of which overlap with or are within protein-coding genes. These results indicate that distinguishing between protein-coding and non-coding RNAs may be difficult in some circumstances (Dinger et al., 2008). Most importantly, Tens of thousands of long ncRNAs have been identified from intergenic regions (lincRNA), as discussed above. More and more lincRNAs have been validated and shown to possess important regulatory functions.

## BROAD FUNCTIONALITY OF LONG ncRNAs

Recent reports have revealed the widespread functionality of long ncRNAs, ranging from epigenetic modification, to transcriptional and post-transcriptional regulation of protein-coding genes. These functions may only account for part of the functional repertoire of long ncRNAs, but they provide quite clear evidence supporting the functional significance of long ncRNAs.

### CHROMATIN MODIFICATION

**FIGURE 2:**
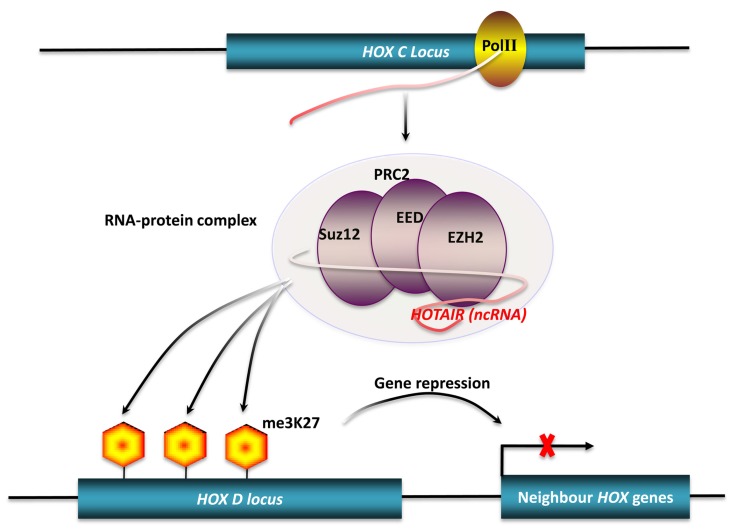
**Chromatin modification by long ncRNA**.

Many studies have shown that long ncRNAs play important roles in chromatin modification (Mattick, 2003; Costa, 2008). Dosage compensation achieved by X-chromosome inactivation (XCI) is a classic example of chromatin modification mediated by long ncRNAs in mammals (Leeb et al., 2009). There are two ncRNAs involved in this process. *Xist*, a 17-kb long ncRNA, initiates XCI, while *Tsix*, an antisense non-coding transcript to the *Xist* gene, opposes XCI. However, the exact mechanism of XCI mediated by these two ncRNAs is still unclear. Ogawa et al. (2008) reported that murine *Xist* and *Tsix* may form *Tsix:Xist* duplexes and be processed into small RNAs by Dicer, then subsequently these small RNAs trigger the RNAi machinery to drive XCI. Another mechanism has been proposed to explain how *Xist* and *Tsix* regulate XCI. In this model, a 1.6-kb ncRNA (*RepA*) transcribed from *Xist* loci identifies and recruits polycomb repressive complex 2 (PRC2), whose catalytic subunit, Exh2, functions as the RNA binding subunit, initiating XCI. *Tsix* keeps the X chromosome active by inhibiting the interaction of *RepA* and PRC2 (Zhao et al., 2008). *HOTAIR* is another well-characterized long ncRNA that can alter chromatin structure by recruiting polycomb proteins. There are 39 human *HOX* genes which can be divided into four clusters (*HOXA-D*) based on their locations on different chromosomes (Woo and Kingston, 2007). A total of 231 *HOX* ncRNAs were identified from these human *HOX* loci. These *HOX* ncRNAs have specific sequence motifs, are spatially expressed along developmental axes, and their expression demarcates broad chromosomal domains of differential histone methylation and RNA polymerase accessibility. A 2.2-kb ncRNA in the *HOX* ncRNA cluster, called *HOTAIR*, can induce heterochromatin formation and repress transcription in *trans* by recruiting PRC2 to trimethylate the lysine-27 residues of Histone H3 in *HOXD* locus (**Figure 2**; Rinn et al., 2007). A common model of epigenetic control relies on ncRNAs acting as chromatin modifying complexes. Another example of this type of mechanism involves the imprinted ncRNA *Air*, which is required for allele-specific silencing of *cis*-linked *Slc22a3*, *Slc22a2*, and *igf2r* genes in mouse placenta. *Air* is believed to target repressive histone-modifying changes by interacting with the *Slc22a3* promoter chromatin and H3K9 histone methyltransferase G9a to epigenetically repress transcription (Nagano et al., 2008). A final example of this type of transcriptional control is driven by *Kcnq1ot1* an antisense ncRNA, that mediates lineage-specific transcriptional silencing patterns by recruiting chromatin-remodeling complexes (G9a and PRC2) to specific regions in the *Kcnq1* locus (Pandey et al., 2008).

### TRANSCRIPTIONAL REGULATION

Many long ncRNAs can directly regulate gene expression at the transcriptional level. Specific mechanisms for direct regulation include transcriptional interference by binding to enhancers, promoters, and transcription factors, the latter being able to alter gene expression at a global level.

**FIGURE 3:**
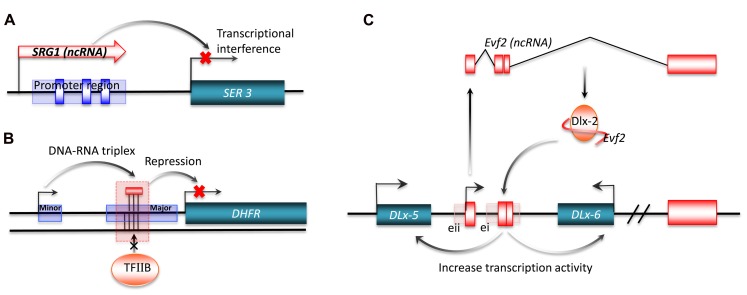
**Transcriptional regulation by long ncRNA.** Long ncRNAs can conduct transcriptional regulation of target genes by: **(A)** transcriptional interference. The purple rectangle represents the promoter region for SER3, and the blue boxes are SER3 upstream activating sequence (UAS) elements; **(B)** interacting with promoters. The short purple rectangle represents the minor promoter, and the long purple rectangle represents the major promoter; **(C)** interacting with transcriptional factors. ei and eii are two ultraconserved enhancers.

Transcriptional interference from long ncRNA has been shown for *SRG1* (*SER3* regulatory gene 1), a well-studied ncRNA in *S. cerevisiae*. The *SER3* gene encodes a serine biosynthesis related enzyme. This gene is strongly repressed and its regulatory region highly transcribed when *S. cerevisiae* are grown in a rich medium. The highly expressed transcript from the SER3 regulatory region was identified by northern blot analysis as *SRG1*, a 550-nt long polyadenylated ncRNA. Substitution analysis of a 150-bp sequence of *SRG1* revealed that *SRG1* can interfere with the activation of the *SER3* promoter to repress *SER3* gene expression (**Figure 3A**; Martens et al., 2004). In metazoa, the *bithoraxoid* (*bxd*) ncRNAs of the fly bithorax complex (BX-C) are a cluster of npcRNAs that have been shown to regulate gene expression by transcriptional interference. In this case, the transcription of several *bxd* ncRNAs are linked to the repression of the *Ubx* (*Ultrabithorax*) protein-coding gene. Transcription of *bxd* ncRNAs represses *Ubx* expression in *cis*, where *Ubx* transcription is repressed by the transcriptional elongation of *bxd* ncRNAs. This is facilitated by the Trithorax complex TAC1, a transcriptional effector that binds to the *bxd* region (Petruk et al., 2006).

Interaction of promoters with long ncRNAs can also regulate gene expression. One example is a non-coding transcript initiated from the upstream minor promoter of the human dihydrofolate reductase (*DHFR*) gene, which represses gene expression by promoter inactivation. The *DHFR* locus has two promoters, with the downstream major promoter responsible for 99% of RNA transcription (Masters and Attardi, 1985). The upstream promoter generates a non-coding transcript that forms a stable complex with the major promoter by interacting with transcription factor II B (TFIIB). This complex acts by dissociating the pre-initiation complex from the major promoter (**Figure 3B**; Martianov et al., 2007). Another signal-induced low-copy-number ncRNA, over 200 nt long, named *ncRNA*_*CCND1*_*s*, also mediates the repression of gene expression by promoter interaction (Wang et al., 2008). *ncRNA*_*CCND1*_*s* recruits a key transcriptional sensor of DNA damage, the translocated in liposarcoma (TLS) RNA-binding protein, to the promoter region of *cyclin D1* (*CCND1*). The recruited TLS RNA-binding protein inhibits the histone acetyltransferase activities of CREB-binding protein (CBP) and p300. This leads to the repression of *CCND1* gene expression in human cell lines. Of particular interest is the signal-induced transcription of *ncRNA*_*CCND1*_*s*, which may provide a novel understanding of stimulus-specific expression of ncRNAs (Wang et al., 2008).

In addition to promoter inactivation or activation, an increasing number of studies now suggest that ncRNAs also regulate gene expression by interacting with transcription factors. One example is *Evf-2*, which is a ~3.8-kb ncRNA transcribed from ei, one of the two *Dlx-5/6* ultraconserved intergenic regions (Zerucha et al., 2000). The ultraconserved region of *Evf-2* specifically interacts with the Dlx-2 protein to form a complex, which increases the transcriptional activity of the *Dlx-5/6* enhancer in a target and homeodomain-specific manner. The stable complex of *Evf-2* ncRNA and the Dlx-2 protein has been validated by *in vivo* assay, indicating that *Evf-2* ncRNA regulates transcriptional activity by directly affecting Dlx-2 activity (**Figure 3C**; Feng et al., 2006). The abundance of such ultraconserved sequences in vertebrate genomes suggests that this mechanism is a common strategy for the regulation of key developmental genes (Bejerano et al., 2004; Sandelin et al., 2004). Another example of this mechanism is *SRA* a ncRNA that interacts with MyoD, a transcription factor that regulates skeletal myogenesis. Through *in vitro* and *in vivo* experiments, Caretti et al. (2006) found that RNA helicases p68/p72, two MyoD-associated proteins, and *SRA* are co-activators of MyoD. The normal activation of muscle gene expression and cell differentiation are suppressed by RNA interference of *SRA*, implying that *SRA* plays an important role in the regulation of developmental gene expression.

Recent experimental evidence has indicated that long ncRNAs could contribute to the complexity of gene expression regulatory networks, where some long ncRNAs might alter global gene expression through a *trans*-acting mechanism. Using gene chip array analysis, Hill et al. (2006) proposed that human introns can coordinate the expression of a wide range of gene products at spatially diverse sites in the genome without miRNAs. Their experiments showed that extensive and specific transcriptional activities in epithelial cells (Hela) were influenced by the expression of three intronic sequences derived from the cystic fibrosis transmembrane conductance regulator (*CFTR*) gene, which was also abnormally expressed. A wide range of genes related to processes of epithelial differentiation and repair were affected as a result of these transcriptional changes, such as FOXF1, sucrase-isomaltase, collagen, interferon, complement, and thrombospondin 1. Hill et al. (2006) suggested that ncRNAs transcribed from intronic regions were responsible for these changes. In a similar vein, the human *Alu* RNA, which is transcribed from short interspersed elements (SINEs), is recognized as a transacting transcriptional repressor which inhibits transcription by binding to RNA polymerase II (Pol II) complexes at promoters *in vitro* as a result of heat shock (Mariner et al., 2008). Because *Alu* elements are so abundant in the human genome, they may contribute to long ncRNA transcriptional repressor function (Amaral and Mattick, 2008).

### POST-TRANSCRIPTIONAL REGULATION

There are many reports providing evidence that ncRNAs have the ability to regulate various aspects of post-transcriptional mRNA processing of, such as splicing, editing, transport, translation, or degradation. The significant role in post-transcriptional regulation of gene expression mediated by small regulatory ncRNAs has been well characterized in various species (see reviews Grishok, 2005; Kavi et al., 2005; Wienholds and Plasterk, 2005; Scherr and Eder, 2007; Filipowicz et al., 2008). Here we discuss how long ncRNAs can mediate post-transcriptional regulation via specific mechanisms.

Some antisense ncRNAs have been shown to regulate gene expression at the post-transcriptional level. For example, *SAF* is a long ncRNA transcribed from the antisense strand of intron 1 of the human *Fas* gene. The overexpression of *SAF* caused the proteins encoded by *Fas* to fail to anchor to the cell membrane and induce *Fas*-mediated apoptosis. It is believed that *SAF* regulates the expression of *Fas* alternative splicing forms through pre-mRNA processing (Yan et al., 2005). Another natural antisense transcript (NAT) of the *Snail1* gene can up-regulate gene expression by forming RNA duplexes in the following fashion. The expression of Zeb2, a transcriptional repressor of E-cadherin, requires an internal ribosome entry site (IRES) derived from a large intron located in the 5′ UTR of the *Snail1* gene, whose expression in epithelial cells triggers an epithelial–mesenchymal transition (EMT). The *Snail1* NAT overlaps with the 5′ splice site of the large intron and Beltran et al. (2008) found that overexpression of this NAT prevented the splicing of the *Zeb2* 5′-UTR, causing an increase in the expression level of the Zeb2 protein. Many antisense transcripts have been mapped to the introns of mammalian genomes (He et al., 2008; Li et al., 2008) indicating that this type of antisense regulation of alternative splicing may be quite common.

**FIGURE 4:**
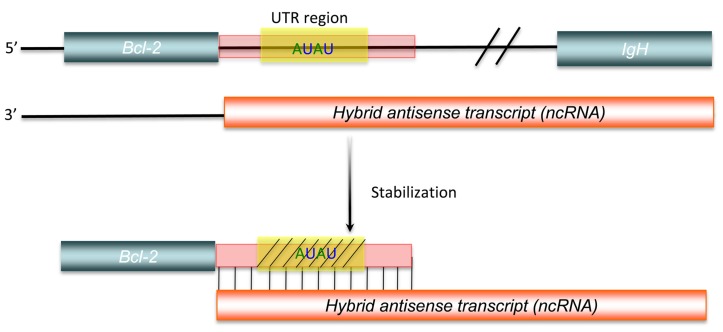
**Post-transcriptional regulation by long ncRNA**.

Another aspect of post-transcriptional regulation of gene expression mediated by long ncRNAs is the stabilization of protein-coding RNAs. Adenylate- and uridylate-rich (AU-rich) elements are specific *cis*-acting elements, found in the 3′ UTRs of many unstable mammalian mRNAs, controlling their half-lives (Bevilacqua et al., 2003). This *cis*-acting regulation can be inhibited, as shown by a *bcl-2*/IgH antisense transcript, formed by with *bcl-2*/IgH translocation, that up-regulates *bcl-2* mRNA expression. This hybrid antisense transcript masks AU-rich motifs present in the 3′ UTR of the *bcl-2* mRNA, increasing the stability of the protein-coding mRNA (**Figure 4**; Capaccioli et al., 1996). Although there is still little direct experimental evidence to identify all mechanisms involved, comparison of genome-scale expression profiles between protein-coding and non-protein-coding RNAs suggests that widespread post-transcriptional control of gene expression via the stabilization of protein-coding RNAs does occur (Nakaya et al., 2007).

## CANCER AND LONG ncRNAs

Many ncRNAs play regulatory roles in cancer biology. Because they regulate cell differentiation and various developmental processes, the mis-expression of long ncRNAs can regulate clinically significant cancer genes.

A number of long ncRNAs have been associated with cancer development and progression. The antisense ncRNA *p15AS* epigenetically silences its sense target gene *p15* in leukemia (Yu et al., 2008). The expression of *p15AS* induces *p15* silencing in *cis* and *trans* through heterochromatin formation. *p15* silencing and increased cell growth were observed after differentiation of mouse embryonic stem cells induced by exogenous *p15AS* (Yu et al., 2008). *ANRIL* (antisense ncRNA from the *INK4A-ARF-INK4B* locus), which is regarded as an isoform of *p15AS*, interacts with chromobox homolog 7 (CBX7), a subunit of the PRC1 protein, and mediates the epigenetic transcriptional repression of its sense locus (Yap et al., 2010). Subsequent study revealed that this ncRNA binds to SUZ12 (suppressor of zeste 12 homolog), a component of the PRC2, and recruits PRC2 to epigenetically repress *p15*^*INK4B*^ (Kotake et al., 2011).

In addition to acting as repressors of tumor suppressor genes, long ncRNAs also contribute to tumorigenesis via other mechanisms. *SRA* is a well-characterized ncRNA, which can co-activate the activity of a number of nuclear receptors in tumors. It can promote muscle differentiation and myogenic conversion of non-muscle cells through the co-activation of MyoD activity as discussed above (Caretti et al., 2006; Hube et al., 2011). Another long ncRNA *PCAT-1* (prostate cancer-associated transcript 1), which is over-expressed in a subset of prostate cancers, particularly metastatic tumors, is known to regulate cell proliferation in prostate cancer progression (Prensner et al., 2011). Moreover, long ncRNA *MALAT1* (metastasis-associated lung adenocarcinoma transcript 1) was shown to be significantly associated with metastasis in non-small cell lung cancer patients (Ji et al., 2003). Subsequent analysis indicated that *MALAT1* was overexpressed in five other non-hepatic human carcinomas (Lin et al., 2007). *MALAT1* may play important roles in tumor cell invasion and formation of metastases (Tseng et al., 2009; Tano et al., 2010). In prostate cancer, a cDNA microarray analysis of intronic transcripts indicated that a high percentage (6.6%) of intronic transcripts were correlated with the degree of prostate tumor differentiation compared to transcripts from unannotated genomic regions (1%; Reis et al., 2004). In renal carcinoma cells (RCC) expression profiles also revealed that there are some non-coding intronic RNAs that are associated with malignant transformation of normal renal cells to tumor cells (Brito et al., 2008). As a result of these and similar observations, long ncRNAs have been used as diagnostic biomarkers because of their cell type-specific or stage-specific expression in different cancers (Mallardo et al., 2008; Reis and Verjovski-Almeida, 2012).

In addition to their functions contributing to tumorigenesis, many ncRNAs are known to act as tumor suppressors. One example is the imprinted gene *MEG3* (maternally expressed gene 3), which functions as a long ncRNA. Although *MEG3* has an open reading frame, it is the folding of *MEG3* RNA that activates p53 expression and selectively regulates p53 target gene expression (Zhou et al., 2007). In addition, *MEG3* can also inhibit cell proliferation via a p53-independent pathway. This evidence suggests that *MEG3* functions as a tumor suppressor in p53 dependent and independent fashion (Zhou et al., 2007; Zhang et al., 2010). Another long ncRNA, *Gas5* (growth arrest-specific 5), binds to the DNA-binding domain of the glucocorticoid receptor (GR), preventing the interaction of glucocorticoid response elements (GRE) with GR. The repression of GR suppresses the glucocorticoid-mediated induction of several genes, leading to apoptosis (Kino et al., 2010). Among the more than 1000 mouse chromatin-state based lincRNAs, one of them (*lincRNA-p21*) functions as a repressor of p53-dependent transcriptional response. *LincRNA-p21* is a transcriptional target gene of p53. It recruits a repressor complex, including heterogeneous nuclear ribonucleoprotein K (hnRNP-K), to a subset of previously active genes, mediating global gene repression and leading to apoptosis (Guttman et al., 2009; Huarte et al., 2010).

These results clearly illustrate the functional significance of long ncRNAs in tumorigenesis and cancer regulatory networks and transcriptional pathways. However, some mechanisms of long ncRNAs in cancer biology seem to be more complicated than expected. For instance, *lincRNA-p21* is transcribed from a region ~15 kb upstream of p21 and mediates apoptosis in a p53-dependent manner upon DNA damage response as discussed above (Huarte et al., 2010). Another single exonic long ncRNA *PANDA* (P21 associated ncRNA DNA damage activated), is transcribed from the ~5 kb upstream region of p21 in an antisense orientation to p21. The expression of *PANDA* is also induced by DNA damage and activated in a p53-dependent manner as *lincRNA-p21*. However, in contrast to *lincRNA-p21*, *PNADA* interacts with the transcription factor NF-YA to limit the expression of some pro-apoptotic genes (Hung et al., 2011). This is just one example of the complexity of cancer-related gene regulation by long ncRNAs. As more long ncRNAs become validated, we can imagine that more regulatory roles of long ncRNAs in tumorigenesis will be unveiled.

## CONCLUSION

The recent explosion in studies of ncRNAs has fostered a new view of the RNA world. It is clear that gene regulation networks are more complicated than expected. And that in future, the central dogma may be challenged by more roles for ncRNAs. Genomes possess a high percentage of non-coding regions, and express a huge repertoire of ncRNAs, which probably contribute to cellular regulatory networks.

The functional significance of ncRNAs has been debated because of their perceived lack of evolutionary conservation. Lower conservation of ncRNAs (mostly for long ncRNAs) was regarded as an argument against functional importance and as a manifestation of transcriptional noise. But less conservation does not mean less function. Many studies indicate that evolutionary constraints on ncRNAs are different to protein-coding RNAs. These different constraints allow many ncRNAs to evolve more quickly subject to positive selection. The complexity underlying the evolutionary conservation of ncRNAs may be stem from the heterogeneity of ncRNAs. ncRNAs derived from different genomic regions may play different regulatory functions. In order to carry out those functions, each class of ncRNA from similar regions may share corresponding specific structures and characteristics, which undergo different evolutionary processes leading to different conservation patterns.

The ncRNA contribution to regulatory networks is complex. Many functional ncRNAs influence chromatin modification, and regulate gene expression at both transcriptional and post-transcriptional levels (Amaral et al., 2010). Although overwhelming evidence has shown that ncRNAs are pervasively expressed from different genomic regions, and possess a wide range of functionality in gene regulation, these discoveries still provide only a glimpse of the hidden ncRNA world. Well-annotated ncRNAs represent a small fraction of the available datasets and the majority of these annotations are structural. While continued advances in high-throughput sequencing will facilitate the discovery and elucidation of more regulatory ncRNAs, we will need a comparable revolution in high-throughput functional testing of ncRNAs to address the functions and mechanisms of long ncRNAs in regulatory networks.

## Conflict of Interest Statement

The authors declare that the research was conducted in the absence of any commercial or financial relationships that could be construed as a potential conflict of interest.
